# Forensic Implications of Anatomical Education and Surgical Training With Cadavers

**DOI:** 10.3389/fsurg.2021.641581

**Published:** 2021-06-23

**Authors:** Carmelo Pirri, Carla Stecco, Andrea Porzionato, Rafael Boscolo-Berto, René H. Fortelny, Veronica Macchi, Marko Konschake, Stefano Merigliano, Raffaele De Caro

**Affiliations:** ^1^Department of Neurosciences, Institute of Human Anatomy, University of Padova, Padova, Italy; ^2^Medical Faculty, Sigmund Freud Private University, Vienna, Austria; ^3^Department of Anatomy, Histology and Embryology, Institute of Clinical and Functional Anatomy, Medical University of Innsbruck, Innsbruck, Austria; ^4^Department of Surgery, Center for Esophageal Disease, Oncology and Gastroenterology, University Hospital of Padova, Padua, Italy

**Keywords:** cadaver lab, medical malpractice, risk management, individual anatomy, iatrogenic lesions, forensic clinical anatomy

## Abstract

Anatomical education and surgical training with cadavers are usually considered an appropriate method of teaching, above all for all surgeons at various levels. Indeed, in such a way they put into practice and exercise a procedure before performing it live, reducing the learning curve in a safe environment and the risks for the patients. Really, up to now it is not clear if the nonuse of the cadavers for anatomical education and surgical training can have also forensic implications. A substantial literature research was used for this review, based on PubMed and Web of Science database. From this review, it is clear that the cadaveric training could be considered mandatory, both for surgeons and for medical students, leading to a series of questions with forensic implications. Indeed, there are many evidences that a cadaver lab can improve the learning curve of a surgeon, above all in the first part of the curve, in which frequent and severe complications are possible. Consequently, a medical responsibility for residents and surgeons which perform a procedure without adequate training could be advised, but also for hospital, that has to guarantee a sufficient training for its surgeons and other specialists through cadaver labs. Surely, this type of training could help to improve the practical skills of surgeons working in small hospitals, where some procedures are rare. Cadaver studies can permit a better evaluation of safety and efficacy of new surgical devices by surgeons, avoiding using patients as ≪guinea pigs≫. Indeed, a legal responsibility for a surgeon and other specialists could exist in the use of a new device without an apparent regulatory oversight. For a good medical practice, the surgeons should communicate to the patient the unsure procedural risks, making sure the patients' full understanding about the novelty of the procedure and that they have used this technique on few, if any, patients before. Cadaver training could represent a shortcut in the standard training process, increasing both the surgeon learning curve and patient confidence. Forensic clinical anatomy can supervise and support all these aspects of the formation and of the use of cadaver training.

## Introduction

For centuries, human cadaveric dissection has been the soul of teaching anatomy (1, 2). Andreas Vesalius, in the sixteenth century, brought cadaveric dissection back to center stage for anatomy students in the University of Padua, influencing education until modern times (3). Being convinced that anatomical knowledge was based on human cadaveric direct observation, he got out of his chair and performed dissections with his own hands (4, 5). While still widely practiced, several practical issues such as availability, cost, and a decrease in anatomy teaching at the undergraduate level made cadaveric training increasingly inaccessible (6). Currently, solving problems involves the use of simulation, the rotations in high-volume centers, and the involvement of multiple trainees in rarely performed procedures (7, 8). However, the gold standard of simulation remains the cadaveric models (9, 10). Several studies have confirmed the utility of fresh frozen cadaver workshops in addressing the challenges in current surgical training, offering a safe, efficient, and effective environment (11). Human body dissection is the most powerful tool to present and learn anatomy, acting also as a dynamic basis for solving problems and being crucial in surgical practice (12). Body dissection is time consuming (13) and does not fit well-with the evident reduction in hours dedicated to learning anatomy (14, 15); for other authors, the costs of maintenance and storage of the cadavers are the disadvantage (13). Cadaver training, in this digital scenario with the rise in the prevalence of informatics, remains the mainstay in anatomy teaching, albeit fueling a continuous debate on its use in teaching (16, 17). Comprehensive software with deep learning and artificial intelligence technologies may have a rising role in medical education, being an integrative tool to combine with the human cadaver dissection (18). Moreover, in the COVID-19 pandemic age, Ross et al. (19) underlined that teaching anatomy with dissection is essential and possible. A crucial challenge is the cadaver availability and accessibility. Infectious diseases, like COVID-19, have increasingly highlighted the importance of cadaver screening before by using established body donation programs, which implies the possibility of accepting or rejecting the donations based on the screening outcomes by mandatory postmortem tests (20). To date, many studies have reported the role of cadaver dissection in anatomical education but also in surgical training, but little attention has been paid to the forensic implications. Really, these topics have essential implications in forensic and medico-legal problems. To highlight the strong relationship among anatomical knowledge, cadaver training, and safe medical practice, the society of forensic clinical anatomy was created in Padova in December 2019. Forensic clinical anatomy is “the part of clinical anatomy (with all its methods and contents) that is useful for legal medicine.” For this reason, forensic clinical anatomy has been illustrated as “the practical application of anatomical knowledge and methods (from ultrastructural to macroscopic aspects), endowed with substantial clinical/surgical implications, to ascertaining and evaluating medicolegal problems” (21). The “methodological definition of Forensic Clinical Anatomy, as a “new” approach to be consistently applied for various anatomical districts, is mainly needed for two reasons: (1) to detail methodological approaches to be followed in the specific cases when anatomy acquires specific forensic relevance; (2) to give new impulse to bio-medicolegal research on the analysis of individual anatomy in the consideration of both clinical/surgical and forensic implications, being quite rare the studies fully addressing the three dimensions of Forensic Clinical Anatomy.”

The “improper performance of surgery,” often for a lack of anatomical knowledge and “improper management of surgical patient” are the two most common allegations when there is a medical malpractice claim (22). Medical responsibility and liability lawsuits have now become a coexistent, albeit inconstant, fact of the professional life of healthcare professionals. The pervasive technological innovation and the hyper-specialization permeating every field of modern medicine imply that each healthcare professional has to chase the edge of knowledge and expertise, to avoid loss of skills and patients or facing malpractice lawsuits (23). On the other hand, the opposite perspective on medical malpractice is given by the positive view offered through the approach of risk management.

The aim of this narrative review was to highlight the implications of cadaveric sessions on surgical training and technical skills performance, in light of the forensic connotations, above all in medical malpractice, where errors may be derived by insufficient anatomical knowledge or surgical training.

## Materials and Methods

**Figure 1:**
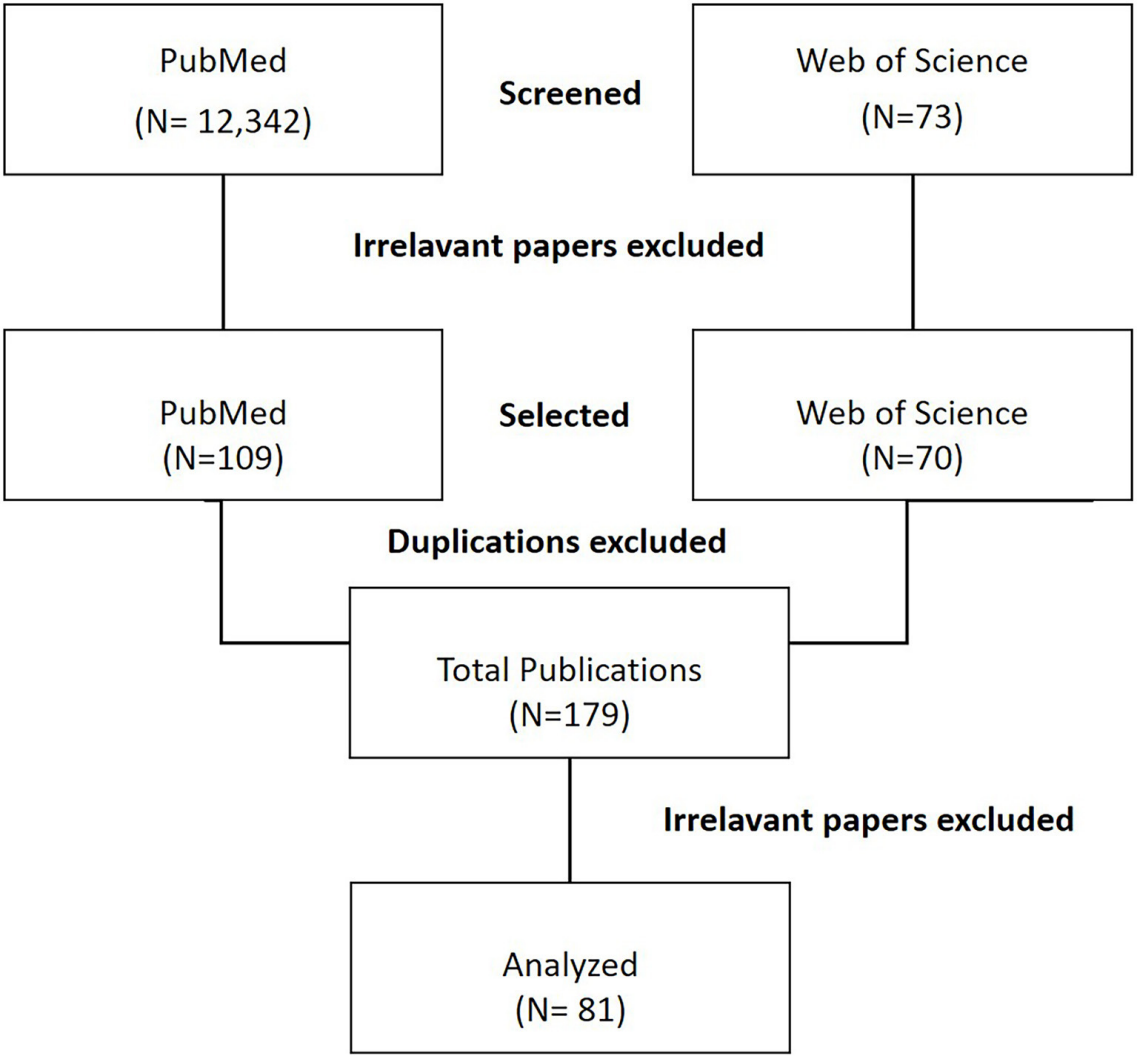
The flowchart of the study.

A substantial literature research was conducted. Preliminarily, in PubMed and Web of Science databases the following search strategy was used: “(anatomy teaching AND forensic implications AND surgical training) OR (cadaver anatomical teaching AND forensic AND surgery) OR (cadaver lab AND forensic AND surgical training) OR (anatomy variations AND surgery AND forensic) OR (anatomy teaching AND surgery AND medical malpractice) OR (dissections AND surgical training AND forensic) OR (anatomy teaching AND risk management AND surgery) OR (anatomy teaching AND device AND surgery) OR (informed consent AND anatomy teaching AND surgery) OR (anatomy AND legal medicine AND surgery).” All types of article were included. Two reviewers (C. Pirri and C. Stecco) independently selected the articles by reading the titles and the abstracts. A third reviewer (A. Porzionato) finalized the selection in case of disagreement. Among the total of 12,415 publications, non-English publications and irrelevant articles were excluded. All data were entered to Endnote software, and duplications were removed. After the selection, each title/abstract (179 publications) was independently evaluated by each of the reviewers/authors and 81 publications were analyzed. All selected papers (81 publications) were critically reviewed and included as appropriate to provide readers with a current overview of research on considerations about forensic implications of anatomical education and surgical training with cadavers (Figure 1). Disagreements were resolved by a consensus. None of the included articles from our research results were assessed for quality or bias using any standard rubrics.

### Purposes of Cadaver Training

**Figure 2:**
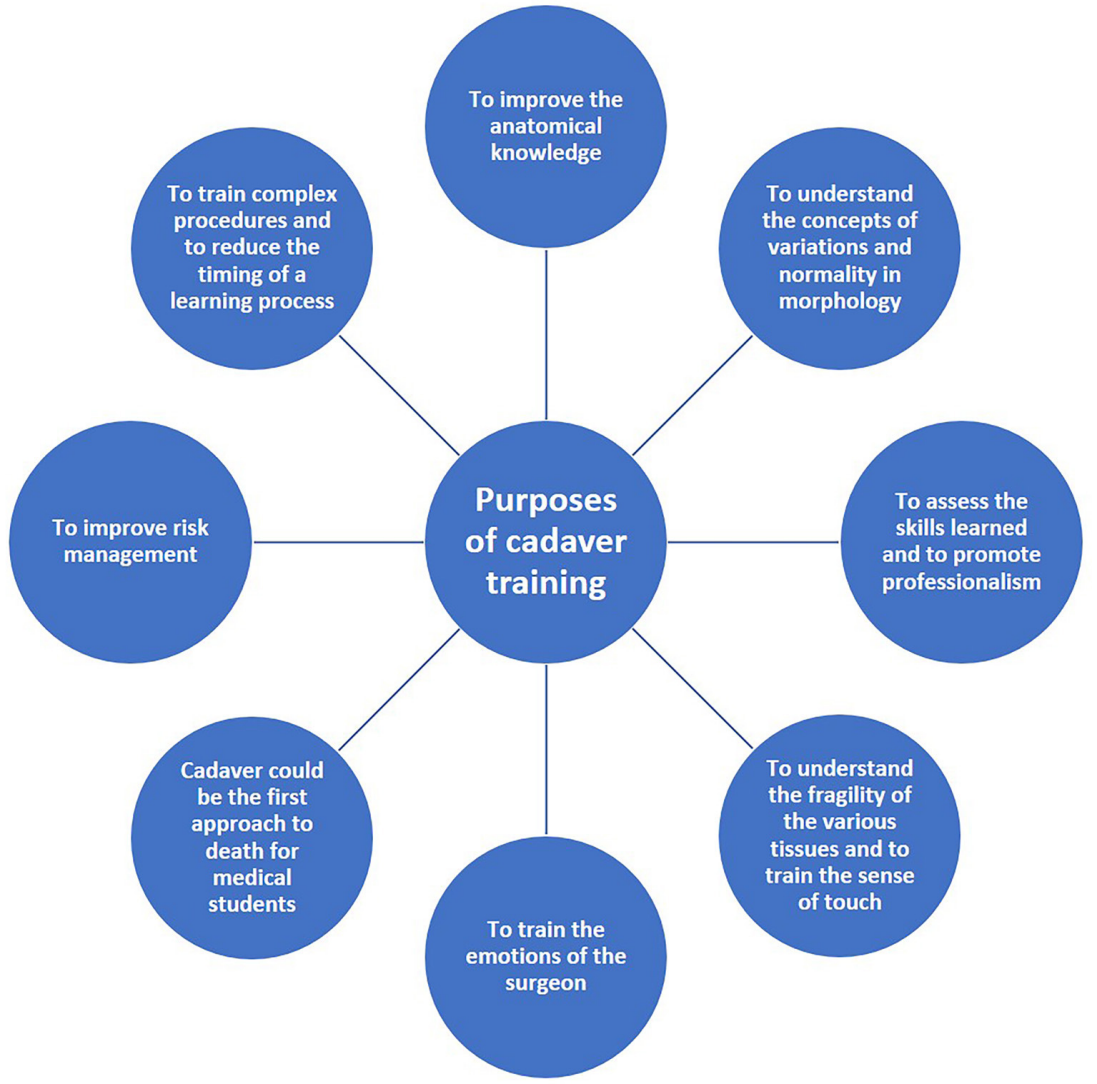
Summary of purposes of cadaver training.

The various purposes of cadaver training are summarized in Figure 2.

### To Improve the Anatomical Knowledge

The understand the human anatomy is a critical component of the practice of medicine. However, a survey by Insull et al. (24) reported that a large part of undergraduates in medicine felt the inadequacy of anatomical knowledge especially from a perspective of safety during medical practice, manifesting the interest in revisiting the dissection of the corpse. Some studies highlighted its role to help students understand the tridimensional relationships among the various anatomical structures (1, 25). Stephens et al. (26) carried out a study to evaluate the role of dissection in anatomical teaching, revealing its role not only for the learning but also for the synergy with the ethical perceptions. Moreover, Snelling et al. (27) reported that the medical students believe that dissection improves their anatomical knowledge but also the capacity to learn other skills such as teamwork, respect for and familiarization with the body, practical skills, and integrating theoretical and practical knowledge. Hamilton and Nagy (28) found that the emergency residents of the second and third years had performances far below those of first-year medical students in anatomy exams. Besides, senior physicians observed that their trainees had poor understanding of the anatomy during the clinical practice (29). Thiels et al. (30) highlighted cases of medical malpractice lawsuits involving surgical residents, underlying some cases in which there were claimed intraoperative injuries like “result of residents becoming anatomically lost without an attending physician present.” Probably one key point is that basic science teaching is often unpopular and boring among preclinical students because they consider irrelevant (31). “The problem is that what may seem irrelevant as a student may become relevant in medical practice” (32). “It is a challenge, therefore, to balance clinical relevance and fundamental basic science concepts in the preclinical curricula” (33). “Clinically integrated and problem-based learning approaches are an attempt to make basic science concepts more relevant for students; however, a major shortcoming of such strategies is inadequate coverage of anatomy” (34). For such reasons, preparatory training courses in topographical anatomy devoted to physicians which later apply to interventional and surgical disciplines are of utmost importance in academic education, improving clinically oriented knowledge, addressing individual interests, and consolidating skills and competences.

According to Hammer et al. (35), it is important to improve the teaching in topographical anatomy directly with cadaver training also for clinical forensic physicians because these lessons produce an important advantage for the safety of the patient, offer the opportunity to steady the skills and competences in a non-judgmental environment, and strengthen the ability of the physician to perform an autopsy lege artis.

### To Understand the Concepts of Variations and Normality in Morphology

Students who can study many specimens or who have the privilege to perform dissections realize the uniqueness of each corpse, and this is in the spirit of forensic clinical anatomy, in which the anatomy of the specific case is assessed in the forensic point of view, highlighting the role of the individual anatomy. The latter “may be defined as the anatomy of that specific person considered in the particular moment of clinical and/or forensic relevance. In this sense, individual anatomy is given by the (statistically) normal anatomy and by all the factors which modify it, i.e., the presence of anatomical variations, age-related modifications (development, maturation, aging) and disease- or surgery-related modifications” (21). Awareness of the relevance of anatomical and clinical individuality is pivotal for the clinician, in assessing diagnostic/therapeutic pathways, and for the medico-legal consultant, in analyzing hypotheses of medical responsibility. The use of plastic models, computer-generated images, simulators, etc., conspires to defer the stage when students encounter anatomical variations and modifications. This trend will compromise the knowledge and understanding of the concept of individual anatomy required to start practicing medicine safely and competently. Moreover, many cadavers are expectedly of the elderly, and it is obvious between anatomists and clinicians. In literature, several morphometric measurements reported are obtained from certain ages, but this is in line with the global life expectancy and consequently with the physiological and pathophysiological changes that it induces, improving the knowledge of the medical doctors (36); they will act above all on this age in daily practice.

One of the principal matters of forensic clinical anatomy investigation is the differential diagnosis, assessing between normal/variant anatomy and pathological/traumatic findings. In literature, several studies reported anatomical variations that had potential forensic pathology implications. Macchi et al. (37), for example, demonstrated that the great variability of the pancreatic arterial vasculature should be carefully considered in a forensic clinical point of view. “Individual anatomy may acquire specific significance in the application of the various steps of analysis in cases of Medical Responsibility and/or Liability” (22). Porzionato et al. (38) highlighted “some cases mainly involving the thyroid, cricoid, and epiglottic cartilages, recently reviewed by Pascual-Font and Sanudo” (39). The differential diagnosis of these variations with foreign bodies or traumatic injuries, for example, owns clinical, surgical, and forensic implications. “Other organ with several anatomical variations and disease/surgery-related modifications is the spleen; all that is known to increase the risk of misdiagnosis, iatrogenic damage and/ or ineffective therapy, for this it is decisive their knowledge by specialists in clinical/surgical disciplines and in legal medicine” (40). Moreover, Macchi et al. (41) showed how the preoperative identification of Brodel's line patterns, during percutaneous and surgical procedures, helps surgeons to minimize hemorrhagic complications requiring an incision of the renal parenchyma in the endophytic renal tumors. “Starting from the considerations that ureteral defect lesions result from retroperitoneal fibrosis, radiation damage, tumors, or surgical procedures and being the management of long-segment ureteral defects a urological challenge, Dal Moro et al. (42) realized a novel surgical procedure (RUG technique, replacement of the ureter with gonadal vein, using the gonadal vein (GoV) as an autologous graft to substitute the ureter.” Anatomical variations lead to potential mistakes which translate to “misdiagnosis (and eventual unnecessary surgery), iatrogenic injury and ineffective therapy.” Regarding the diagnosis, characteristic aspects and assessments should be investigated focusing on “the anamnesis (i.e., previous surgery or trauma ecc.), physical examination, choice and analysis of instrumental exams.” However, even if it is true that some anatomical situations could also escape to a detailed and careful evaluation before the surgical act, it is also true that the variations and modifications of a particular anatomy of an organ, a nerve, a blood vessel, etc., must carefully be evaluated first.

### To Train Complex Procedures and to Reduce the Timing of a Learning Process

In the last decade, a steady increase in the number of students admitted to university courses for the health professions was recorded, with a corresponding reduction in per capita training resources, also in the context of the study of human anatomy. This phenomenon also involved specialist medical training, in which during the postgraduate training there is an increase in the responsibilities assumed which goes hand in hand with the technical and executive skills required (43). Moreover, the demand for less invasive surgical approaches by healthcare institutions, industry, and patients is increasing. Nevertheless, the development of a specific training approach is required for the safety in the use of new techniques and technologies into clinical practice. “Surgical training naturally includes vocational training with patients in an operating room, but the reduction in working hours, decreased operating time, and ethical imperatives to protect patients have all resulted in a decrease in hands-on experience” (11). Eckert et al. (44) hypothesized “that the increasing use of non-operative management, percutaneous and endoscopic intervention, minimally invasive surgery, and endovascular surgery has radically altered case mix and resident training. This may radically change the abilities and expectations for the field of general surgery and what it means to be a general surgeon.” Indeed, the exposure in preparation to the surgical task is the most challenging element of many complex open procedures, in addition to the performance of the task. “The 2010 National Vascular Resident Report indicates minimal experience for vascular surgical trainees in complex vascular exposures. Despite this, the end product of training is still expected to manage vascular trauma or iatrogenic injuries to blood vessels” (45).

There is no doubt that the learning exercise may involve the commission of errors, which if made on patients imply possible medical-legal disputes (22, 46). Consequently, it is necessary that the physician in training—but the same goes for health professionals more generally—can practice safely to acquire the skills required also for basic procedures, for example by using cadaver labs. Calio et al. (47) demonstrated that the residents acquire satisfaction, confidence, and perception of the patient safety by the supervised cadaver training, prior to the initiation of spine surgical rotation. Many different cadaver trainings were proposed to improve this lack of knowledge. Làszlò et al. (48) highlighted the teaching of fibreoptically guided intubation of the trachea by the use of Thiel's cadaver. Tomlinson et al. (49) affirmed that both the “Thiel and Crosado cadavers offered lifelike simulation of pedicle screw insertion, with each having advantages depending on whether the focus is on soft tissue approach or technical aspects of bony screw insertion in the spinal surgery.” Eljamel et al. (50) evaluated “the use of Thiel cadaveric model to practice MRI-guided deep brain stimulation (DBS) implantation and high-frequency MRI-guided high frequency focused ultrasound (FUS) in the human brain.” Lone et al. (51) showed that Thiel-embalmed cadavers provided a useful tool for simulation of a local anesthetic injection technique. Porzionato et al. (52) demonstrated that Thiel cadavers are suitable for laparoscopy and also for procedures of the Natural Orifice Transluminal Endoscopic Surgery (NOTES), whose development and learning are usually realized with the swine models and fresh frozen cadavers. Surely, this training could be expensive, but it reduces the risk for the patients. Besides, in this setting it is also possible to integrate/replace the use of the whole body with selected anatomical parts for specific needs or human remains recovered from surgical amputations, having a training that it will not necessarily be a money-consuming process (46, 53). Furthermore, Balta et al. (54) underlined the decrease in the infectious health risks associated with handling human cadavers, by the use of the different embalming solutions. Moreover, Venne et al. (55) demonstrated that also other types of embalming such as phenol-based offers a realistic model for surgical training skills, preserving the integrity of the skin. Balta et al. and Noël et al. (54, 56) suggested that “the new skill-centered curricula which require residents to perform specific tasks within realistic settings, exhibit a growing demand for life-like cadaveric specimens,” underlining that “Institutions' body donation programs must, therefore, adapt to those greater need for cadaveric specimens, which presents many challenges, ranging from the logistical to the ethical.”

In various surgical fields, the decision about what type of surgical approach to be used (for instance, open surgery, endovascular, laparoscopic) also depends on the specific experience of the staff. This aspect is considered in various guidelines and also invests the medico-legal judgment in matters of liability. The availability of a body donation program for surgical training may permit to expand the capability to apply different approaches, with positive implications in terms of professional responsibility.

### To Understand the Fragility of the Various Tissues and to Train the Sense of Touch

For a surgeon, the understanding of the fragility of the various tissues with touch is a key point. Therefore, improving the sense of touch of surgeons, many simulators are equipped with handles endowed of haptic feedback to simulate these sensations. Actually, in a survey of Vapenstad et al. (57), only 5% of the surgeons confirmed that the friction in the simulator's handles reproduced the reality (5%), while 95% considered it too high. Consequently, the surgeons of this study “preferred the handles without haptic feedback because they perceived the handles with haptic feedback to add additional friction, making them unrealistic and not mechanically transparent.” LeBlanc et al. (58) compared a fresh cadaver with an advanced laparoscopic simulator used to learn sigmoid colectomy with laparoscopic hand assistance and reported that the cadaver model is far superior regarding anatomic replication and realism, suggesting that it better replicates the conditions of dexterity to manage the intestinal tissues and the planes of dissection. The roles of models and simulators are limited by the lack of realism and poor representation of human anatomy.

Also, animal models are often considered a good alternative to cadaver to understand the tissue texture and planes of gliding. They have several limitations: for example, the swine is the model with a shape of the chest more similar to humans, but having differences in its morphology is hardly usable to simulate the chest compressions. The comparability of the models is invalidated and restricted due to differences of the global cardiopulmonary physiology. Pelvic surgery is also very different in pigs with respect to humans. Moreover, it is important to stress that the possibility to use living animal models for surgical training is very limited nowadays on the basis of concerns about animal welfare (59, 60).

### To Train the Emotions of the Surgeon

The surgical room can be stressful for inexperienced residents, and it can be a source of high anxiety levels. Cadaver training is a worthwhile and efficacious tool in the education of surgeons, not only to decrease their anxiety level but also for the perception of self-care and well-being. According to the study of Daly et al. (61), “subjective stress levels in the wet laboratory are higher (32.4%) compared with the dry laboratory (5.4%, *p* < 0.001). In addition, more students felt that the wet laboratory prepared them for the anxiety (55.4 vs. 24.3%, *p* < 0.001) and technical demands (67.6 vs. 44.6%, *p* = 0.005) of the operating room.”

In a study by Romo-Barrientos et al. (62), the emotional anxiety of the students, “measured as state anxiety, dropped from 14.7 to 10 points (*p* < 0.05) after cadaveric training.” These students found that their experiences in the dissecting room can upset their emotional balance. Besides, for the authors, the implementation of coping mechanisms could be a very effective strategy to reduce their anxiety and also to improve their learning outcomes, helping to strengthen their practical skills. Thus, beneficial effects even in error prevention may be hypothesized, this aspect needing further analyses in the future.

### Cadaver Could Be the First Approach for Medical Students to the Death and the Assessment of the Skills Learned, Promoting Professionalism

The ethical aspects related to patient care and death are a fundamental part of the medical training, but it is often forgotten, and the students are not used to a correct approach toward death. Cadaver labs help the medical students toward progressive contact with a dead body in a safe and tranquil environment. According to the study of Goss et al. (63), the students involuntarily alternated perceptions of the corpse as a learning specimen or feel the cadaver as a person, with a penchant for one or the other. The “specimen” view trained the technical part approach to dissection, while the “person” view is the emotional one. This is a crucial moment in the training, in which anatomical experience puts together the medical activity by the students, connecting it to their different professional developments. “Specimen-minded students intentionally objectified the body to learn the emotional control physicians need, while person-minded students humanized the body donor to promote the emotional engagement required of physicians” (63). The two aspects of the approach promote respectively the emotional control and the emotional engagement required to a physician.

Besides, the cadaver is often considered by the students as their “first patient.” This perspective has the primary purpose of promoting principled habits in medical students, but according to Cohen (64), this could create a didactic situation that leads to an opposite result. The danger of considering the dissection subject as lifeless without participation discourages the emotions and promotes untested coping mechanisms to the clinical situation, “values like detached concern, a controversial practice in medicine can be implicitly present.” Probably, it could be better to consider the cadaver as a “silent mentor,” showing the body donor's deeds and attitudes toward life and death when they were alive. Besides, to reinforce the humanity and to increase the learning aptitudes of the medical students, it is important to introduce an initiation ceremony in the medical curriculum before approaching the cadaver. Chiou et al. (65) demonstrated that this ceremony increases the students' attitudes toward death and decreases the negative emotions toward cadavers. Besides, emotions that are being respected, cherished, and appreciated are significantly higher in those students.

Professionalism is one of the six areas of competency defined by the Accreditation Council for Graduate Medical Education (ACGME). Unprofessional behavior is the single most common cause for disciplinary action against medical students in their clinical rotations, residents, and clinical practitioners (66). According to Escobar-Poni and Poni (66), cadaver laboratory dissection has a crucial position, “being the basis of several activities where professional behaviors can be taught, practiced, and rewarded, as demonstrate respect, compassion, and integrity, demonstrate sensitivity and responsiveness patient's culture, age, gender, and disabilities and maintain appropriate relations with patients in every circumstance.”

Besides, Sargent Jones et al. (67) showed that the cadaver-based practical exams are the best tool to enable students to understand and learn the three-dimensional gross medical anatomy.

All the above considerations are strictly connected with the bioethical and deontological issues of medicine, as usually addressed in medico-legal contexts.

### Cadaver Training and Risk Management

The clinical risk management is aimed at improving the quality and safety of healthcare services by identifying the conditions that put patients and personnel at risk of harm and then acting accordingly to prevent and control those risks (68).

Moreover, traditionally the teaching of new surgical procedures for the operators is performed as a combination of clinical observation, tutor instruction, and supervised procedures in real patients. This methodology of teaching ensures daily experience, which can be messy when there is the competition among various trainees for the same experience within a unique hospital. For example, the rate of major bleeding complications requiring blood transfusion or radiological or surgical intervention after renal biopsy in literature varies between 0.4 and 8%, and these different rates are related with the number of procedures done in various centers. Therefore, does a hospital with 8% of major complications have a risk management problem? If the hospitals have the possibility to reduce the complication rate with cadaveric training, has this training been mandatory? Undoubtedly, the patients find these differences unacceptable. Before a surgeon achieves competence in a new procedure, there is a phase of learning where the procedure is considered physically and mentally demanding, and the operation takes longer and often results in poor results with a higher complication rate and less efficacy (69, 70). For example, the incidence of bile duct injury during laparoscopic cholecystectomies is 2.2% in the first 13 patients, while it decreases to 0.1% in subsequent patients (71). For carotid angioplasty, the risk of patients to incur a death/stroke is 8% at the beginning of the learning curve, while this rate declines to 0% over the 40-month period (72). According to a survey of the Vanderbilt University Center Emergency department (2,000) about the willingness of patients to participate in training on specific procedures, only 47% of them will be available to be the first patient in which a resident could perform simple procedures like lumbar puncture, intubation, or sutures, and surely this percentage will decrease in case of more complex operations (73). For this reason, often, the residents do not explain to the patients that they are physicians in training (74). This is an unethical behavior; according to the “Council on Ethical and Judicial Affairs of the American Medical Association,” the training status of the medical doctors and residents is considered a critical data during the signature of the informed consensus. Indeed, an informed consent includes three components: capacity, voluntariness, and information, the latter involving instructions on risks (also about the physician's experience performing the procedure) and benefits, which are the three main pillars on which the informed consent is based. “It is possible that the risk of complications may be greatest when the physician's experience performing the procedure is minimal. Besides, inexperience increases the potential for discomfort because the procedure may take longer” (75).

A similar problem is present also for surgeons involved in new procedures. Indeed, “only one third of them specifically mention the innovative nature of the anticipated operation during the consent process” (76), also if the safety and efficacy of these new techniques have not been established with sure evidence (77). “The ethical boundaries of using patients as ≪guinea pigs≫ in order to refine innovative surgical skills are indistinct and, in practice, remain largely a matter of personal, professional conscience” (77). Therefore, to prevent risks to patients, control and regulation would be necessary. To reduce this problem, in the Institute of Human Anatomy of the University of Padova thanks to the body donation program (20), the surgeons are supported to plan a new surgical approach respecting all the ethical boundaries thanks to a full integration of anatomical, radiographic, and clinical perspectives (78–80).

## Conclusion

Anatomical education and surgical training with donated bodies are pivotal for all surgeons, from junior trainees to experienced, allowing them to practice a procedure before performing it live and to make mistakes in a safe environment. This training is mandatory, leading to a series of questions with forensic implications: could there be medical liability for a resident or a surgeon who performed a procedure without a proper training? Knowing that a cadaver lab can improve the learning curve of a surgeon, above all in the first part of the curve, where there are the most frequent and severe complications, has this training become mandatory for a surgeon? Besides, should a hospital provide a sufficient training for its surgeons and other specialists, maybe through cadaver labs? Surely this type of training could help to improve the practical skills of surgeons working in small hospitals, where some procedures are rare. Finally, could a legal responsibility exist for a surgeon and other specialists who use a new device without an apparent regulatory oversight? Cadaver studies can permit a better evaluation of safety and efficacy of new surgical devices by surgeons, avoiding to use patients as ≪guinea pigs≫. For a good medical practice, the surgeons should inform the patient about the uncertain risks associated with the procedure, making sure the patients' full understanding about the novelty of the procedure and that they have used this new technique on few, if any, patients before. Cadaver training could represent a shortcut in the standard training process, increasing both the surgeon learning curve and patient confidence. The forensic clinical anatomy can supervise and support all these aspects of the formation and of the use of cadaver training.

## Author Contributions

CP, CS, AP, VM, and MK conceived the study and co-wrote the paper. CP, CS, AP, RB-B, RHF and MK extracted all data. CP, CS, AP, VM, SM, and RD undertook and refined the search. RB-B, VM, SM, and RD helped to revise the intellectual content. All authors contributed to the article and approved the submitted version.

## Conflict of Interest

The authors declare that the research was conducted in the absence of any commercial or financial relationships that could be construed as a potential conflict of interest.
